# Baicalin Weakens the Virulence of Porcine Extraintestinal Pathogenic *Escherichia coli* by Inhibiting the LuxS/AI-2 Quorum-Sensing System

**DOI:** 10.3390/biom14040452

**Published:** 2024-04-08

**Authors:** Bingbing Zong, Yong Xiao, Peiyi Wang, Wei Liu, Mingxing Ren, Changyan Li, Shulin Fu, Yanyan Zhang, Yinsheng Qiu

**Affiliations:** 1 Hubei Key Laboratory of Animal Nutrition and Feed Science, Wuhan Polytechnic University, Wuhan 430023, China; zongbingbing@whpu.edu.cn (B.Z.);; 2 Engineering Research Center of Feed Protein Resources on Agricultural By-Products, Ministry of Education, Wuhan Polytechnic University, Wuhan 400023, China; 3 Hubei Collaborative Innovation Center for Animal Nutrition and Feed Safety, Wuhan 400023, China

**Keywords:** porcine ExPEC, baicalin, quorum-sensing system, LuxS/AI-2, virulence, PCN033

## Abstract

Porcine extraintestinal pathogenic *Escherichia coli* (ExPEC) is a pathogenic bacterium that causes huge economic losses to the pig farming industry and considerably threatens human health. The quorum sensing (QS) system plays a crucial role in the survival and pathogenesis of pathogenic bacteria. Hence, it is a viable approach to prevent ExPEC infection by compromising the QS system, particularly the LuxS/AI-2 system. In this study, we investigated the effects of baicalin on the LuxS/AI-2 system of ExPEC. Baicalin at concentrations of 25, 50, and 100 μg/mL significantly diminished the survival ability of ExPEC in hostile environments and could inhibit the biofilm formation and autoagglutination ability in ExPEC. Moreover, baicalin dose-dependently decreased the production of AI-2 and down-regulated the expression level of *luxS* in PCN033. These results suggest that baicalin can weaken the virulence of PCN033 by inhibiting the LuxS/AI-2 system. After the gene *luxS* was deleted, AI-2 production in PCN033 was almost completely eliminated, similar to the effect of baicalin on the production of AI-2 in PCN033. This indicates that baicalin reduced the production of AI-2 by inhibiting the expression level of *luxS* in ExPEC. In addition, the animal experiment further showed the potential of baicalin as a LuxS/AI-2 system inhibitor to prevent ExPEC infection. This study highlights the potential of baicalin as a natural quorum-sensing inhibitor for therapeutic applications in preventing ExPEC infection by targeting the LuxS/AI-2 system.

## 1. Introduction

*Escherichia coli* (*E. coli*) is a bacterial species that can cause various types of infections. One specific type is extraintestinal pathogenic *E. coli* (ExPEC), which possesses certain virulence factors [1]. ExPEC infections can progress from the intestines to other organs, leading to various disorders [2,3] and, consequently, substantial financial losses [2]. Moreover, ExPEC is resistant to multiple drugs and is associated with a number of diseases in both humans and animals [4,5,6]. It can invade the bloodstream, resulting in septicemia [7]. Numerous studies have revealed similarities in the serogroups and pathogenicity characteristics of porcine and human ExPECs, suggesting the potential for porcine ExPEC to spread to humans [8,9]. Recently, the segregation rate of porcine ExPEC has increased due to the rapid growth of the porcine industry, leading to the emergence of multiple-drug-resistant *E. coli,* which further complicates the mitigation and control of porcine ExPEC infections [10,11]. Currently, there are no effective preventative measures or therapies for porcine ExPEC infections, making it crucial for their effective prevention and control to investigate effective therapeutic medications.

Quorum sensing (QS), a form of cell–cell communication, involves the generation and emission of QS signaling molecules, followed by their binding to receptors and the stimulation of downstream signals [12]. Such communication enables microbial populations to adapt to environmental changes at the community level. Pathogenic bacteria, including *E. coli*, use QS as a regulator for various biological processes such as biofilm formation, the production of secondary metabolites, and interactions with hosts and other microorganisms. In particular, QS plays a crucial role in the generation of virulence factors and the development of antibiotic resistance [13]. The AI-2, one of the QS signaling molecules, is produced by both Gram-positive and Gram-negative bacteria and is involved in the regulation of multiple bacterial processes [14]. The LuxS, an enzyme, plays a role in the synthesis of AI-2 by catalyzing the formation of homocysteine (Hcys) and 4,5-dihydroxy-2,3-pentanedione (DPD) from S-ribosylhomocysteine (SRH), whereas DPD undergoes spontaneous cyclization to form AI-2 [15]. The LuxS/AI-2 system, found in a large proportion of Gram-positive and Gram-negative bacteria, functions as an important global bacterial regulator by using AI-2 as an indicator molecule. It modulates bacterial population dynamics by regulating gene transcription and influencing cell behavior [12,16,17]. Quorum-sensing inhibitors (QSIs) have gained significant attention in the field of anti-infection research and development. These compounds can increase the susceptibility of bacterial biofilms to antimicrobial agents and reduce bacterial virulence without affecting their growth and development. This potentially mitigates the development of drug resistance [18].

**Figure 1:**
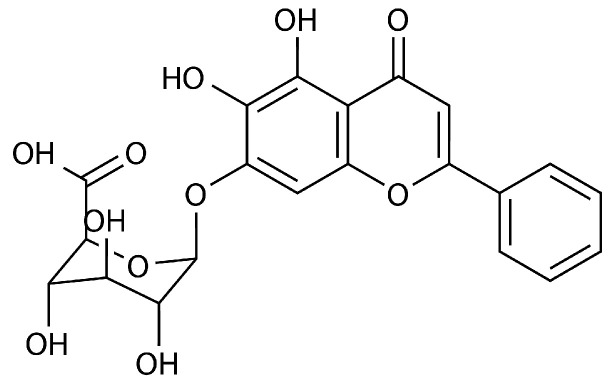
The structure of baicalin.

The antibacterial properties of chemicals originating from plants have been studied extensively. Some of the primary sources of natural QSIs are the roots, rhizomes, flowers, and leaves of plants [19]. Baicalin (BA; Figure 1), a principal pharmacological component found in *Scutellaria radix*, a plant of the family Lamiaceae, has been documented in the Chinese, European, and British Pharmacopoeias and has a wide range of therapeutic uses and pharmacological actions, including anticancer, antibacterial, and oxidative effects [20,21]. In addition to these properties, BA also inhibits bacterial DNA, RNA, and protein production and breaks down endotoxins to exert its antibacterial effects [22]. In a recent study, BA attenuated QS-controlled virulence and enhanced *Pseudomonas aeruginosa* clearance in a mouse peritoneal implant infection model [23]; it also inhibited QS against avian pathogenic *Escherichia coli* (APEC) [24]. However, there are no reports on the use of BA as a QSI in the treatment of porcine ExPEC.

In this study, we aim to provide a novel approach for the prevention and treatment of porcine ExEPC and the development of BA as a QSI. We present evidence that BA reduces the virulence of porcine EXPEC in a mouse model, which is achieved by influencing the production of AI-2 signaling molecules in the LuxS/AI-2 system.

## 2. Materials and Methods

### 2.1. Strains, Growth Conditions, and Reagents

The ExPEC PCN033 strain used in this study was first isolated from a farm in Hunan China in 2006 and was donated by the Key Lab of Preventive Veterinary Medicine in Hubei Province [10]. *Vibrio harveyi* (*V. harveyi*) BB170, which was used for the bioluminescence assay for AI-2, was donated by Huazhong Agriculture University and stored in our laboratory. The mutant ∆*luxS* and the complemented strain C∆*luxS* were constructed in this study. The DH5α strain was obtained from Vazyme (Nanjing, China). The strains and plasmids mentioned in Table 1 and the primers mentioned in Table 2 were used in this study. The ExPEC strains were cultivated in Luria–Bertani (LB) broth medium (Qingdao Hope Bio-Technology Co., Ltd., Qingdao, China, HB0128) in a shaker at 37 °C for 180 rpm. Strain *V. harveyi* BB170 was cultured using autoinducer-bioassay (AB) medium (Shandong Tuopu Biol-Engineering Co., Ltd., Qingdao, China, M2289) at 30 °C. Baicalin (CAS:21967-41-9, purity ≥ 98%) was acquired from Sichuan Xieli Pharmaceutical Co., Ltd. (Pengzhou, China).

### 2.2. Cell Culture

Porcine kidney cells (PK-15), which were stored at our laboratory, were cultured in DMEM/High glucose medium (Cytiva, Shanghai, China) supplemented with 10% fetal calf serum, 100 U/mL penicillin, and 10 mg/mL streptomycin sulfate. The cells were incubated at 37 °C in a humidified 5% CO_2_ incubator.

### 2.3. Construction and Identification of Mutant and Complemented Strain

The suicide plasmid pRE112 was used to construct the mutant by homologous recombination as described previously [25], with some modifications. Briefly, the upstream (LU/567 bp) and downstream (LD/581 bp) homologous arms of gene *luxS* were amplified by *luxS*-u-F/R and *luxS*-d-F/R. Subsequently, overlapping extension PCR was performed to obtain a fused homologous arm (UD), which was transferred into the suicide plasmid pRE112 with the restriction enzymes SacI and XbaI to construct the recombinant plasmid pRE112-UD. The recombinant plasmid was then introduced into the *χ*7213 and cultured in an LB nutrient agar (Qingdao Hope Bio-Technology Co., Ltd., Qingdao, China, HB0129) plate with 50 μg/mL chloramphenicol (Sigma, Shanghai, China) to pick up the recombinant strains (wild-type strains with suicide plasmid pRE112). The recombinant strains were then simultaneously cultured in LB medium with or without chloramphenicol, and we validated those strains that showed no growth in chloramphenicol but in LB by the internal and external source primers to obtain the mutant strains.

The construction of complemented strains was performed as described previously [25], with some modifications. Briefly, we used the wild-type strains as templates to amplify the gene *luxS* by the primers C*luxS*-F/R to obtain the fragment with restriction enzymes XbaI and KpnI. This fragment was then inserted into the plasmid pHSG396, and the reaction product was transferred into a mutant to obtain the complemented strains. Validation of the complemented strains was performed using the primers VF/R.

### 2.4. Growth Curve Analysis

To determine the effect of baicalin on the growth characteristics of PCN033 after deletion of the gene *luxS*, the overnight-activated strains were transferred at a ratio of 1:1000 to fresh LB medium and incubated for 4 h in a shaker at 37 °C for 180 rpm. After dilution to the appropriate gradient, the bacterial numbers were determined for each hour.

The OD_600_ value was detected using the Microbial Growth curve analyzer (Ningbo Scientz Biotechnology Co., Ltd., Ningbo, China, MGC-200) as recommended by the manufacturer. The overnight-activated strains were diluted at a ratio of 1:1000, and 200 μL of diluted bacterial solution was added into a 96-well plate. The conditions were as follows: a temperature of 37 °C, 1100 rpm for pre-shaking, and 800 rpm for culture, with a sampling interval of 1 h to read the OD_600_ value.

### 2.5. Gene Expression Assay

To determine the transcriptional level of ExPEC PCN033, we used reverse transcription-quantitative polymerase chain reaction (RT-qPCR) after incubation with BA (25, 50, 100 μg/mL) for 4 h. The total RNA was extracted using a bacterial RNA extraction kit (Vazyme, Nanjing, China) following the manufacturer’s instructions, and 1 μg of total RNA was reverse-transcribed by the ABScript Neo RT Master Mix for qPCR with gDNA Remover (ABclonal, Wuhan, China). The Q-PCR reactions were performed in a QuantStudio™ 1 Plus Real-Time PCR System (ThemoFisher, Shanghai, China) with BrightCycle Universal SYBR Green qPCR Mix with UDG (ABclonal, Wuhan, China).

### 2.6. AI-2 Activity Assay

To determine the ability to produce AI-2 of PNC033, the AI-2 bioassay was performed as described previously [26,27], with some modifications. Briefly, after overnight activating and transferring at a ratio of 1:1000 into fresh LB medium, followed by incubation with BA (25, 50, 100 μg/mL) for 4 h at 37 °C in a shaker at 180 rpm, we collected the supernatant of the strains after centrifugation at 4 °C with 12,000 rpm for 15 min. After filtering through 0.22 μm filters, the samples were stored at −20 °C. *Vibrio harveyi* BB170, which is an AI-2 reporter strain, was diluted at a ratio of 1:5000 in fresh AB medium, and 180 μL of BB170 culture mixed with 20 μL of the previously supernatant was added to an opaque 96-well plate (Corning Costar, Cambridge, MA, USA) and incubated at 30 °C for 4 hours. Reading was performed using a SpectraMax^®^ i3x (Molecular Devices, LLC, Shanghai, China) in luminescence mode. *Vibrio harveyi* BB170 was used as positive control. The measure of AI-2 activity was performed as described above, but the supernatant was collected from 0 to 8 h.

### 2.7. Biofilm Formation Assay

The biofilm formation assay was performed as described elsewhere [24,27,28]. After activation overnight in LB, the strains were transferred at a ratio of 1:1000 with 50 μL of diluted bacterial suspension into a 96-well plate (Corning Costar, Fisher Scientific, Canada), spiked with 150 μL of BA (25, 50, 100 μg/mL), and incubated at 37 °C for 24 h. The supernatant was removed using sterile water and washed twice. Methanol was used to fix the biofilm for 15 min. After that, 0.1% crystal violet was applied for staining, and after 15 min, the sample was washed twice with sterile water. Finally, we applied 33% Hac to dissolve the remaining crystal violet and read the OD_570_ value.

### 2.8. Adherence and Invasion Assay

The adherence and invasion assays were performed as described previously [24,25], with some modifications. Briefly, confluent monolayers of PK-15 cells (10^6^ cells/well) were incubated with BA (25, 50, 100 μg/mL) and PCN033, with a multiplicity of infection (MOI) of 100 for 1.5 h at 37 °C with 5% CO_2_. Subsequently, the monolayers were washed with phosphate-buffered saline (PBS) twice, and 200 μL of pancreatin (Gibico, Shanghai, China) was added to co-culture the cells for 5 min and isolate them from the wells. Finally, 800 μL of PBS was used to resuspend the cells, followed by dilution to a suitable gradient to determine the bacterial counts on an LB agar plate.

Unlike the adherence assay, the invasion assay was performed as follows. After incubation with BA (25, 50, 100 μg/mL) and PCN033 for 1.5 h, the monolayers were washed with PBS twice, then subsequently incubated for 1 h with cefotaxime acid (Aladdin, Shanghai, China) to clean up those bacteria that did not invade the cells. Subsequently, the monolayers were washed with PBS twice, and 200 μL of pancreatin (Gibico, Shanghai, China) was added to co-culture the cells for 5 min and isolate them from the wells. Finally, 800 μL of PBS was used to resuspend the cells, followed by dilution to a suitable gradient to determine the bacterial counts on an LB agar plate.

### 2.9. Stress Resistance Assay

The stress resistance assay was conducted as described in a previous study [27,29], with some modifications. The strains were initially activated overnight in LB medium and then diluted with BA (25, 50, 100 μg/mL) in fresh LB medium. The cultures were maintained at 37 °C, 180 rpm, for 4 h. After the incubation period, for the heat resistance assay, 1 mL of the bacterial fluid was pipetted into an Eppendorf tube and soaked in water at 48 °C for 1 h. Simultaneously, the bacterial fluid was placed in a water bath heated to 37 °C as a control. In the hyperosmotic stress assay, 500 μL of the bacterial fluid and 500 μL of 2 M potassium chloride solution (Sinopharm, Beijing, China) were pipetted into an Eppendorf tube. The control group was treated with 500 μL of PBS instead of potassium chloride. The tubes were then incubated in a shaker at 37 °C, 180 rpm, for 60 min. Finally, the bacterial counts (colony-forming units/mL, CFU/mL) were determined using dilution separation methods. The survival rate was calculated as follows:

Survival rate (%) = (CFU_stress group_)/(CFU_control group_) × 100% (1)

### 2.10. Whole-Blood Bactericidal Experiments

Whole-blood bactericidal experiments were conducted as described elsewhere [25], with slight modifications. Briefly, whole blood was collected from the swine’s jugular vein into a vacutainer (Jiangsu Kangjie Medical Devices Co., Ltd., Taizhou, China, KG092K2E). After activation by overnight culturing in LB medium, the bacterial suspension was co-incubated with BA (25, 50, 100 μg/mL) in a shaker at 37 °C, 180 rpm, for 4 h. Subsequently, the bacteria were collected by centrifugation at 12,000 g for 5 min; the supernatant was removed and resuspended in PBS until reaching 10^8^ CFU/mL. After this, the bacterial suspension and the whole blood were mixed in a ratio of 1:9 and co-cultured upside down at 37 °C for 3 h. Bacterial suspension mixed with PBS was considered as control. Finally, the mixture was kept on ice for 10 min to terminate the reaction, and the bacterial count was determined via dilution separation. The survival rate was calculated as follows:

Survival rate (%) = (CFU_blood group_)/(CFU_control group_) × 100% (2)

### 2.11. Autoagglutination Assay

The autoagglutination assay was performed as described elsewhere [30,31], with some modifications. First, after overnight activation by culturing in LB medium, the bacterial strains were transferred at a ratio of 1:1000 into fresh LB medium and co-cultured with BA (25, 50, 100 μg/mL) in a shaker at 37 °C 180 rpm for 4 h. Second, the bacterial suspension was centrifuged at 7000 rpm for 5 min and then resuspended with PBS to adjust the OD_600_ to the same value. Third, the bacteria were divided into two 15 mL centrifugal tubes and kept at room temperature (25 °C). One tube was considered as a test group and kept on the shelf, and the other tube was the control group, which was vortexed before measuring. The autoagglutination levels were determined using the following equation:

Autoagglutination (%) = (OD_600_ value of the test group)/(OD_600_ value of the control group) × 100% (3)

### 2.12. Animal Experiments

All animal experiments were approved by the Scientific Ethics Committee of Wuhan Polytechnic University under permit number WPU202307001.

The female Kunming mice (4–6 weeks) were purchased from the Center for Disease Control of Hubei Province (Wuhan, China). The mouse infection model PCN033 was used according to a previous study [25]. The dosage of BA in vivo was selected based on previous studies performed by our group [32,33,34], with some modifications.

Mouse protection assay: Fifty mice were randomly divided into the following five groups of ten mice each: Group A: PBS (intraperitoneal injection, IP) + PBS (intramuscular injection, IM); Group B: PCN033(IP)+PBS(IM); Group C: PCN033(IP) + 50 mg/kg·bw BA(IM); Group D: Δ*luxS* (IP) + PBS(IM); Group E: CΔ*luxS*(IP) + PBS(IM). The bacterial strains were cultured overnight and then diluted 1000 times with fresh LB medium, followed by culturing in a shaker at 37 °C, 180 rpm, for 4 h and adjusting the concentration with PBS to 10^7^ CFU/mL. The initial administration of BA occurred simultaneously with the establishment of PNC033. The BA was administered twice daily, and mortality was recorded for 14 days.

Mouse anti-infection assay: Thirty mice were randomly divided into five groups of six mice each, and the grouping and BA administration were performed as described in the mouse protection assay. After 6 h of infection, the mice were anesthetized with Zoletil^®^ 50, and then blood was collected retro-orbitally. Subsequently, the mice were euthanized by cervical dislocation, and the brain, heart, liver, spleen, lung, and kidney were separated. General-purpose tissue fixer (Servicebio, Wuhan, China, G1101) was used to fix parts of the brain, heart, liver, spleen, lung, and kidney. The remaining tissue was homogenized with 1 mL of PBS, and the CFU value was determined via dilution separation.

### 2.13. Statistical Methods

Comparisons between the two groups were made using the unpaired Student’s two-sided *t*-test, and one-way analysis of variance (ANOVA) was performed for more than three groups. * *p* < 0.05, ** *p* < 0.01, *** *p* < 0.001, **** *p* < 0.0001.

## 3. Results

### 3.1. BA Weakened the Survival Ability of PCN033 under Adverse Environmental Conditions

**Figure 2:**
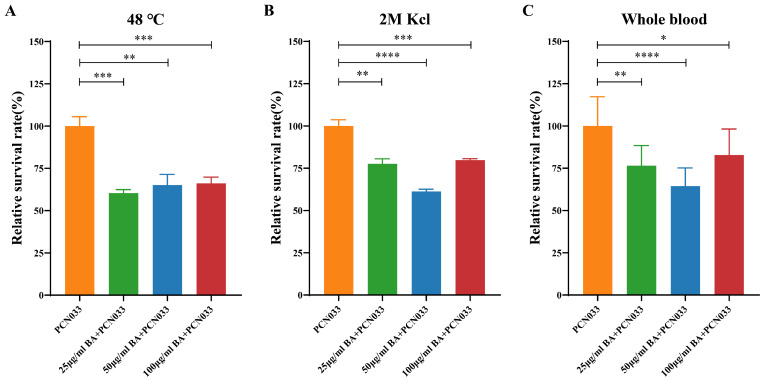
Effects of BA on the survival ability of PCN033 in a hostile environment. (**A**) Effects of BA on the survival rate of PCN033 at 48 °C. (**B**) Effects of BA on the survival rate of PCN033 in a highly concentrated potassium chloride solution. (**C**) Effects of BA on the survival rate of PCN033 in whole blood. Statistical significance was assessed by one-way ANOVA compared to that of the PCN033 group (* *p* < 0.05, ** *p* < 0.01, *** *p* < 0.001, **** *p* < 0.0001).

In our previous study, the minimum inhibitory concentration (MIC) of BA on PNC033 was >1600 µg/mL [32], and BA at 100 μg/mL had no effect on the growth characteristics of PCN033. In this study, we investigated the effect of BA on the survival ability of PCN033 in hostile environments. As shown in Figure 2, BA (25, 50, and 100 µg/mL) significantly reduced the survival rate of PCN033 at high temperature (Figure 2A), at high osmotic pressure (Figure 2B), and in whole blood (Figure 2C).

### 3.2. BA Weakened the Survival and Pathogenesis of PCN033 by Inhibiting the LuxS/AI-2 System

**Figure 3:**
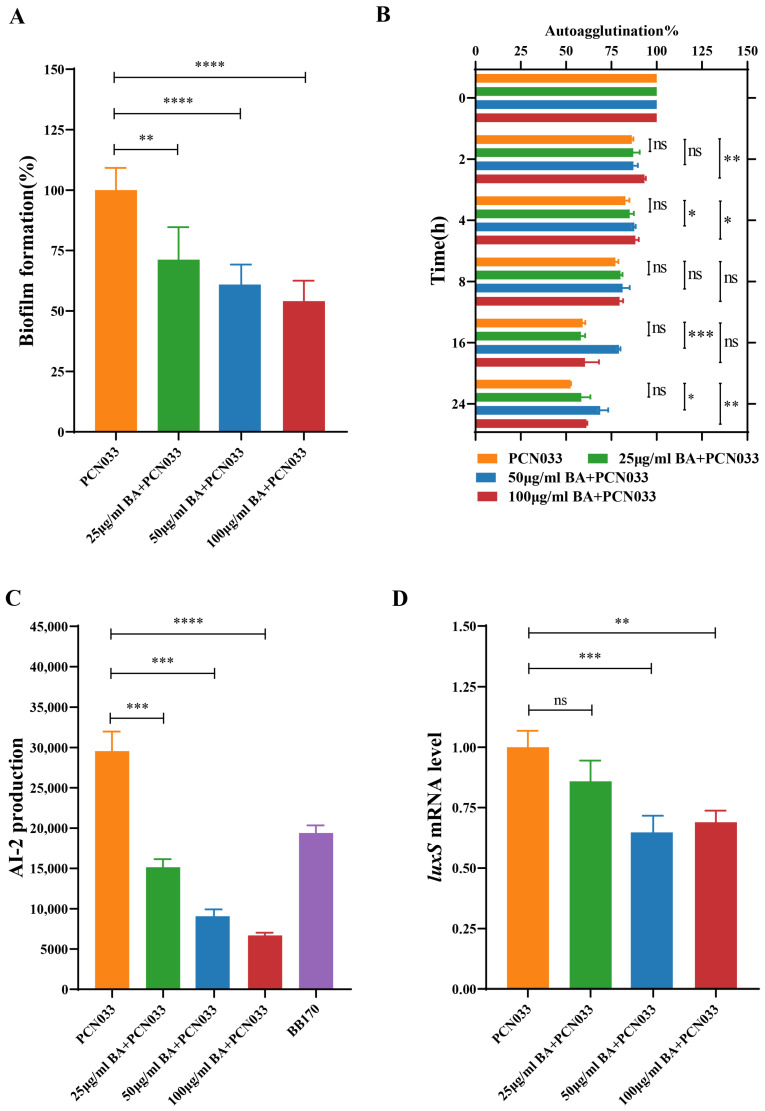
BA affects the virulence of PCN033 by inhibiting the LuxS/AI-2 system in vitro. (**A**) Effects of BA on the biofilm formation of PCN033. (**B**) Effects of BA on the autoagglutination of PCN033. (**C**) Effects of BA on the AI-2 production of PCN033. (**D**) Effects of BA on the mRNA expression level of the gene *luxS* in PCN033. Statistical significance was assessed by one-way ANOVA compared to that of the PCN033 group (* *p* < 0.05, ** *p* < 0.01, *** *p* < 0.001, **** *p* < 0.0001, ns means no significance).

Combining the biological function of biofilm as mentioned in the previous paragraph, the above findings (Figure 2) suggested that BA might have an effect on the biofilm formation of PCN033. Therefore, we further evaluated the effects of BA on the biofilm formation of PCN033. Based on the results (Figure 3A,B), BA significantly decreased the biofilm formation of PCN033 in a dose-dependent manner and significantly slowed down the autoagglutination rate of PCN033 in the treated groups after 16 h. Further analysis revealed that BA significantly reduced AI-2 production and down-regulated the gene expression of the *luxS* gene in PCN033 (Figure 3C,D). Based on these findings, we hypothesize that BA reduces PCN033 biofilm formation by inhibiting the LuxS/AI-2 system, consequently limiting its survival in hostile environments.

### 3.3. BA Reduced the Activity of AI-2 by Inhibiting the Expression of the Gene luxS

**Figure 4:**
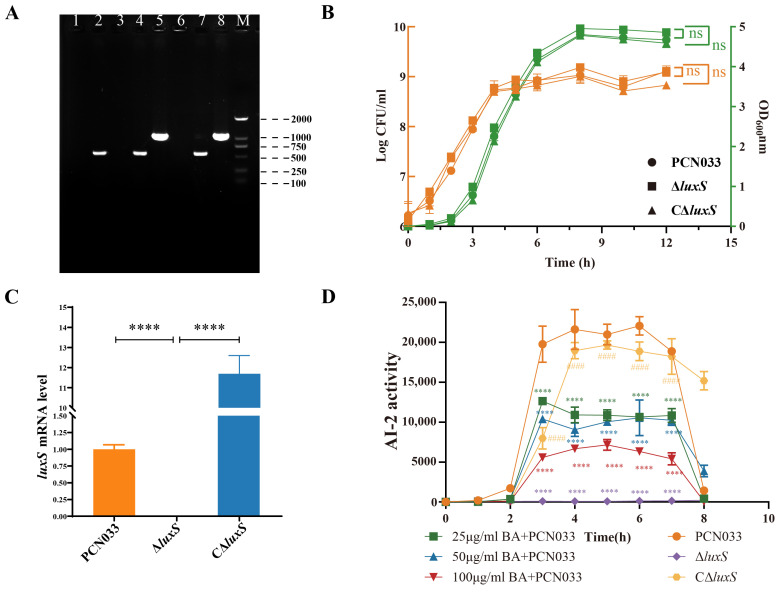
BA reduces the activity of AI-2 by inhibiting the expression level of the gene *luxS*. (**A**) PCR validation of the ∆*luxS* and C∆*luxS*. Lane 1: ∆*luxS* (Internal source primers), Lane 2: PCN033 (Internal source primers), Lane 3: Negative control (Internal source primers), Lane 4: ∆*luxS* (External source primers), Lane 5: PCN033 (External source primers), Lane 6: Negative control (External source primers), Lane 7: C∆*luxS* (VF/R), Lane 8: pHSG396(VF/R), M: DNA marker (DL2000). (**B**) Growth characteristics of ∆l*uxS* and C∆*luxS*. (**C**) mRNA levels of gene *luxS* in the ∆*luxS* and C∆*luxS* strains. (**D**) Effects of BA and *luxS* deletion on the AI-2 activity of PCN033. Statistical significance was assessed by one-way ANOVA compared to that of the PCN033 group (**** *p* < 0.0001, ns means no significance). Statistical significance between ∆*luxS* and C∆*luxS* was assessed by an unpaired Student’s two-sided *t*-test (#### *p* < 0.0001).

Figure 4A shows that the mutant ∆*luxS* and its complemented C∆*luxS* strains were successfully constructed. The growth curve (Figure 4B) showed that the deletion of *luxS* does not affect the growth characteristics of PCN033. The expression level of the gene *luxS* of Δ*luxS* was significantly down-regulated, and the C∆*luxS* restored the expression level (Figure 4C). As shown in Figure 3C,D and Figure 4D, BA inhibited AI-2 production during PCN033 growth, and the ∆*luxS* strain no longer produced AI-2, indicating that BA reduced AI-2 production by affecting the transcript level of *luxS*.

### 3.4. Decrease in AI-2 Activity, Caused by BA, Inhibited the Expression Level of luxS and Decreased the Survival Ability of PCN033 In Vitro

**Figure 5:**
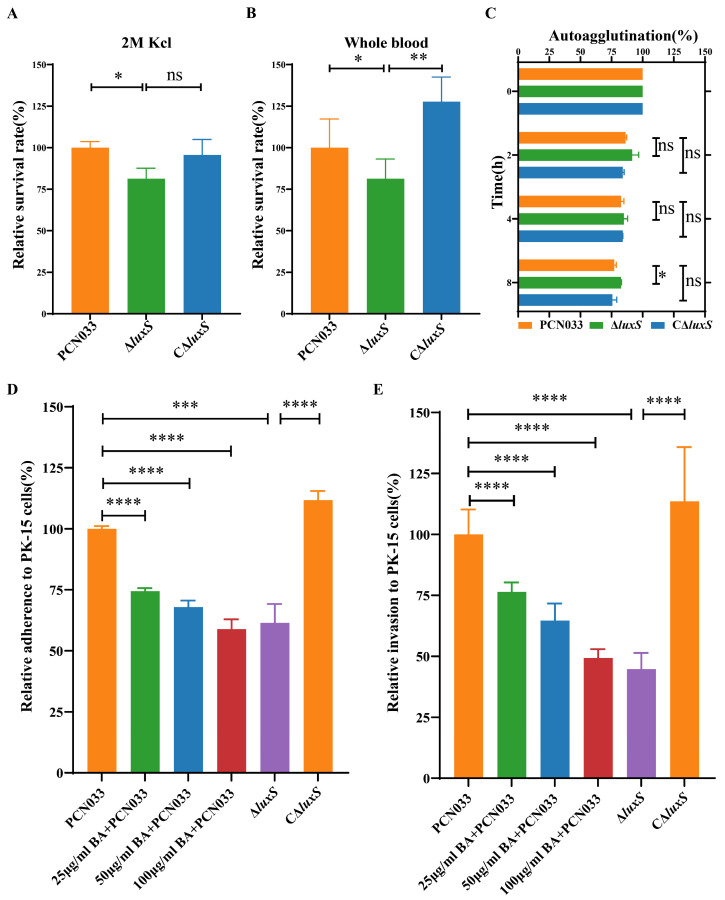
Effects of the decreased activity of AI-2 caused by BA on the pathogenesis and survival of PCN033 in vitro. (**A**) Survival rates of ∆*luxS* and C∆*luxS* in a highly concentrated potassium chloride solution. (**B**) Survival rates of ∆*luxS* and C∆*luxS* in whole blood. (**C**) Autoagglutination of ∆*luxS* and C∆*luxS*. (**D**) Effects of BA and *luxS* deletion on the ability of PN033 to adhere to PK-15 cells. (**E**) Effects of BA and *luxS* deletion on the ability of PN033 to invade PK-15 cells. Statistical significance was assessed by one-way ANOVA compared to that of the PCN033 group, and statistical significance between ∆*luxS* and C∆*luxS* was assessed by an unpaired Student’s two-sided *t*-test (* *p* < 0.05, ** *p* < 0.01, *** *p* < 0.001, **** *p* < 0.0001, ns means no significance).

The survival rate of the ∆*luxS* strain exhibited a significant reduction in the highly concentrated potassium chloride solution (Figure 5A) and in whole blood (Figure 5B). The autoagglutination (Figure 5C) of PCN033 was also attenuated. To further understand the effect of BA on the virulence of PCN033, an adherence and invasion assay was performed. The results (Figure 5D,E) showed that gene *luxS* deletion and co-incubation with BA (25, 50, 100 µg/mL) yielded similar results, significantly reducing the ability of PCN033 to adhere or invade PK-15 cells. Thus, we conclude that BA weakens the virulence of PCN033 in vitro by inhibiting the LuxS/AI-2 system.

### 3.5. The Decrease in AI-2 Activity Caused by BA Inhibited the Expression Level of luxS and Decreased the Pathogenesis Ability of PCN033 In Vivo

**Figure 6:**
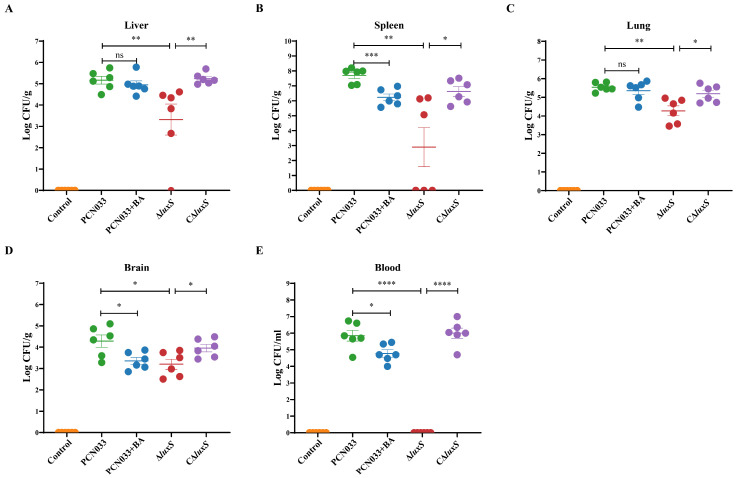
Effects of BA and *luxS* deletion on the ability of PCN033 to colonize mice. (**A**) Effects of BA and *luxS* deletion on the bacterial load in the liver. (**B**) Effects of BA and *luxS* deletion on the bacterial load in the spleen. (**C**) Effects of BA and *luxS* deletion on the bacterial load in the lung. (**D**) Effects of BA and *luxS* deletion on the bacterial load in the brain. (**E**) Effects of BA and *luxS* deletion on the bacterial load in the blood. Statistical significance was assessed by one-way ANOVA compared to that of the PCN033 group, and statistical significance between ∆*luxS* and C∆*luxS* was assessed by an unpaired Student’s two-sided *t*-test (* *p* < 0.05, ** *p* < 0.01, *** *p* < 0.001, **** *p* < 0.0001, ns means no significance).

**Figure 7:**
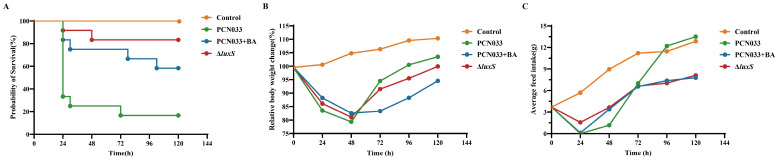
Effects of BA and *luxS* deletion on the virulence of PCN033 in mice. (**A**) Effects of BA and *luxS* deletion on the survival rate of mice infected with PCN033. (**B**) Effects of BA and *luxS* deletion on the body weight of mice infected with PCN033. (**C**) Effects of BA and *luxS* deletion on the average daily feed intake (AFDI) of mice infected with PCN033.

To further evaluate the effects of the decreased production of AI-2 caused by BA on the pathogenesis ability of PCN033, the mouse infection experiment was performed. The mice were infected with PCN033 and then treated with 50 mg/kg·bw BA. The bacterial loads in the liver, spleen, lung, brain, and blood of the BA group showed a significant reduction compared to those observed for the PCN033 group. Similarly, the bacterial loads in the liver, spleen, lung, brain, and blood of the ∆*luxS* group also showed a significant reduction compared to those of the PCN033 group (Figure 6). The survival curve (Figure 7A) demonstrated that both deleting the *luxS* gene and treatment with BA significantly reduced the mortality rate of the infection. Additionally, the weight curve and average daily feed intake (ADFI) curve (Figure 7B,C) indicated that within 48 h after infection, the weight and ADFI of mice were significantly reduced compared to those of the control group. Importantly, the ∆*luxS* group and the BA treatment group (BA group) showed a slower reduction in weight and ADFI compared to the PCN033 group. These findings suggest that BA has a protective effect against PCN033 infection by inhibiting the LuxS/AI-2 system, which impairs the virulence of the bacteria.

**Figure 8:**
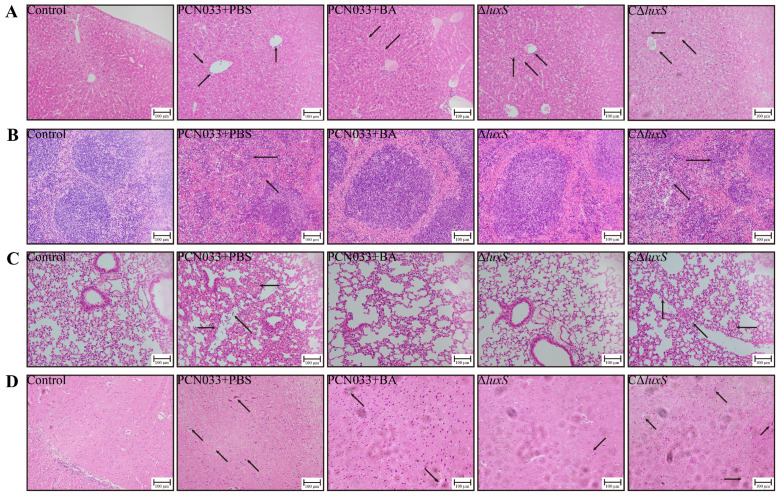
Effects of BA and *luxS* deletion on the histopathology of PCN033 infections in mice. (**A**) Histopathological changes in the HE staining of the liver. (**B**) Histopathological changes in the HE staining of the spleen. (**C**) Histopathological changes in the HE staining of the lung. (**D**) Histopathological changes in the HE staining of the brain. Histopathological changes are indicated by arrows. Scale (black line): 100 μm. The dosage of BA was 50 mg/kg∙bw.

The histological analysis presented in Figure 8 revealed distinct alterations in various organs of the mice. In the liver (Figure 8A), disorganization and partial edematous changes were observed in the hepatocytes of the PCN033 group and the CΔ*luxS* group. These alterations were accompanied by loose cytoplasm. In the spleen (Figure 8B), significant degeneration of splenic corpuscles and an increase in megakaryocytes were noted in the PCN033 group and the CΔ*luxS* group. The lung exhibited thickened alveolar walls, inflammatory cell infiltration, and a small amount of hemorrhage in the PCN033 group and the CΔ*luxS* group (Figure 8C). In the brain (Figure 8D), the cells showed loose cytoplasm and an enlarged gap, with an increased number of vacuolated cells and some broken nerve fibers, all observed in the PCN033 group and the CΔ*luxS* group. Importantly, these histological changes were notably alleviated in both the BA-treated group and the Δ*luxS* group (Figure 8A–D). These observations strongly suggest that BA administration is an effective treatment modality for reducing PCN033 infections by affecting the bacterium’s virulence.

## 4. Discussion

Baicalin, a flavonoid with multiple pharmacological effects such as anti-inflammatory, antibacterial, and antioxidant activities [20,35], has proven antimicrobial properties [23,24,36,37]. However, the effect of BA on porcine ExPEC, a bacterial strain that can cause extraintestinal infections, resulting in significant economic losses in animal husbandry [38,39], has been poorly investigated. In our previous study, we observed that BA’s minimum inhibitory concentration (MIC) for PCN033 was over 1600 μg/mL [32]. In the present study, we explored the effects of BA on the virulence of PCN033 and its protective effects on mice following infection with PCN033.

Given that the ability of bacteria to adjust to modifications in the environment is crucial for their survival, microbes have developed several adaptation strategies for stress. Bacteria unavoidably adapt to a variety of stressful circumstances when they grow. For example, *E. coli* may experience a variety of stressful situations whilst surviving in the intestines of humans, including food scarcity, hyper/hypo-osmolarity, low pH, and oxidants [40]. This capacity allows them to survive under stress, facilitates the cultivation of intricate groups, and activates pathogenicity reactions following host infection. Mihaljevic et al. [41] observed that subjecting *Campylobacter jejuni* isolates to temperatures between 42 and 55 °C for 3 min reduced their cultivability and survivability as well as impaired adhesion and invasion efficiency. In the present study, the survival rate of bacteria co-cultured with BA was significantly reduced under different conditions such as a temperature of 48 °C, a 2 M potassium chloride solution, and a whole-blood environment (Figure 2). Biofilm, which is primarily composed of autogenic extracellular polymeric substances, plays a crucial role in holding bacteria collectively on surfaces, protecting them, preventing phagocytosis, and enabling colonization and persistence over time [42]. It is closely associated with the pathogenicity of bacteria, with 65–80% of infections being biofilm-related [43]. To further investigate the effects of BA on PCN033, we assessed the biofilm formation of PCN033 and found that BA markedly reduced biofilm formation (Figure 3A). Autoagglutination, which involves non-adherent bacteria attaching themselves to adhesive ones, is believed to promote colonization, improve the infectious dosage per particle, and provide protection against adverse environmental conditions [30]. In *Yersinia enterocolitica*, *Y. pestis*, and *Y. pseudotuberculosis*, only strains with autoagglutination capacity are considered to be virulent [44]. In our study, the autoagglutination ability of the BA treatment group was significantly weaker than that of the control group after 16 h or more of settling (Figure 3B). These results suggest that BA affects bacterial virulence.

Quorum sensing regulates various bacterial activities such as pathogenicity, competency, combination, antimicrobial synthesis, mobility, spore production, and biofilm formation [12,45]. Generally, LuxS is responsible for synthesizing AI-2, a signaling molecule that senses population density in the LuxS/AI-2 quorum-sensing system. The AI-2 is synthesized from S-ribosyl homocysteine (SRH) through the actions of LuxS [15,46]. In this study, we evaluated the effect of BA on the LuxS/AI-2 system and its impact on the virulence of PCN033; based on the results, BA significantly inhibits the production of AI-2 (Figure 3C). To further investigate the mechanism, we examined the transcript levels of *luxS* and observed a significant reduction in *luxS* expression with BA treatment (Figure 3D). Based on the environmental stress conditions (Figure 2), biofilm formation (Figure 3A), and autoagglutination (Figure 3B), these findings suggest that BA weakens the survival ability of PCN033 in vitro via the LuxS/AI-2 system.

To investigate the impact of reducing AI-2 on the survival ability of PCN033, we constructed the *luxS* gene deletion mutant and its complementary strains. The PCR validation confirmed the successful construction of the *luxS* gene deletion mutant and its complementary strains (Figure 4A). The generation of virulence factors and the development of biofilms are regulated by QS, and the most prevalent bacterial autoinducer reported yet, AI-2, is synthesized by the enzyme *luxS*, which converts SRH into the highly active compound 4,5-dihydroxy-2,3-pentanedione (DPD), which then autonomously cycles into AI-2 [16]. The signal transmission cascade which results in a synchronized gene expression in the bacterial population is activated once the environmental quantity of AI-2 exceeds an upper limit [14].

Therefore, the amount of AI-2 is significant in the LuxS/AI-2 QS system. As shown in Figure 3D, BA down-regulates the gene expression of *luxS*. Moreover, when treated with BA, there is a significant reduction in AI-2 production during the entire growth process, and the deletion of the *luxS* gene completely abolishes AI-2 production in the bacteria, as observed during the growth period (Figure 4D). Consequently, the ability of PCN033 to resist environmental stress is considerably reduced upon *luxS* deletion (Figure 5A,B). These findings align with the results of Zhang et al. [27] who reported a weakened stress resistance in the Δ*luxS* mutants of *Haemophilus parasuis* (*H. parasuis*). Furthermore, the absence of *luxS* also reduces the ability of autoagglutination (Figure 5C), as observed in previous studies [27,47]. Adherence is the initial step for bacteria to interact with cells, and our study illustrates that BA significantly reduces the adherence and invasion ability of PCN033 to PK-15 cells; similarly, the adherence and invasion ability of Δ*luxS* was also compromised in our study (Figure 5D,E), consistent with previous findings [27,48,49]. In conclusion, BA exhibits the potential to reduce the virulence of PCN033 through the LuxS/AI-2 system.

To further validate the role of BA in weakening the virulence of PCN033 through the LuxS/AI-2 system, a mouse model of PCN033 infection was used. Stroeher et al. [50] evaluated the virulence of Δ*luxS* mutant strains compared to that of wild-type strains by using an input ratio of approximately 3:1. After 24 h, no mutant strains were detected in the blood or spleen. This finding is consistent with the results of our whole-blood bactericidal test (Figure 5B) and the tissue bacterial load test (Figure 6), where the deletion of *luxS* resulted in reduced bacterial survival in whole blood. At 6 h after the intraperitoneal injection of PCN033 into mice, the bacterial loads of the Δ*luxS* group in the heart, liver, spleen, lung, kidney, brain, and blood were significantly lower than those of the wild-type group. Similar results were observed when treating PCN033 with BA. This indicates that deficiency of *luxS* or treatment with BA is unfavorable for the survival of PCN033 in the host. In *Edwardsiella piscicida* (*E. piscicida*), the Δ*luxS* mutants exhibited weaker lethality against zebrafish compared to the wild type [51]. In a mouse model using *luxS* mutation in *Streptococcus pneumoniae*, the survival time of the mutant group was significantly longer than that of the wild-type group [50]. The Δ*luxS* mutants of *Streptococcus agalactiae* also displayed lower virulence in tilapias compared to the wild type [49]. This leads us to infer that the deletion of *luxS* reduces the virulence of bacteria, as demonstrated in our study (Figure 7A), where the Δ*luxS*-deleted strains of PCN033 exhibited a lower mortality rate in the mouse infection model. Furthermore, during the reinfection period, mice infected with the Δ*luxS* strain exhibited less weight loss (Figure 7B) and higher food consumption (Figure 7C) in comparison to those infected with the wild-type strain. Moreover, the histopathological changes (Figure 8) in mice infected with the Δ*luxS* strain were mitigated compared to those infected with the wild-type strain. Notably, similar trends were observed in the BA-treated group, as evidenced by Figure 6, Figure 7 and Figure 8, providing further evidence for the notion that BA impairs the virulence of PCN033 through the LuxS/AI-2 system.

**Figure 9:**
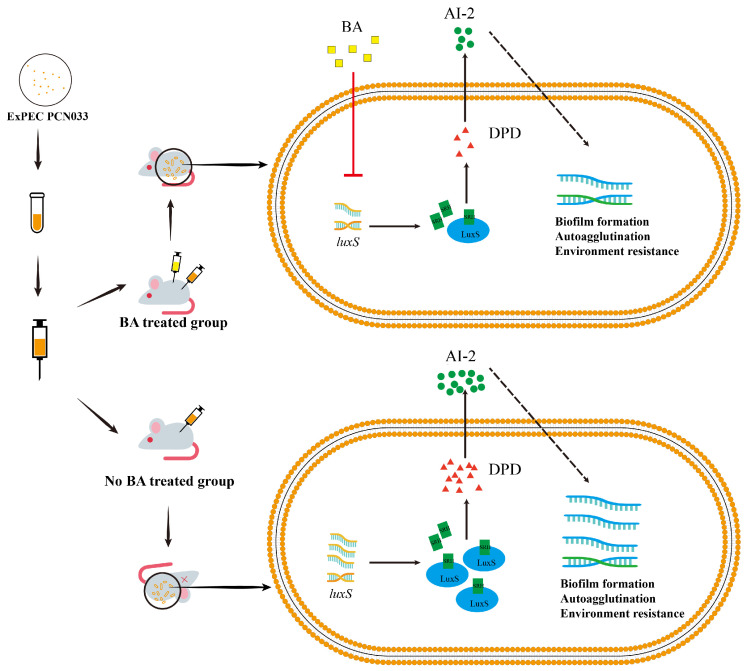
The mechanism of BA weakens the survival and pathogenesis of ExPEC by inhibiting the LuxS/AI-2 system. ExPEC, extraintestinal pathogenic *Escherichia coli*; BA, baicalin; DPD, 4,5-dihydroxy-2,3-pentanedione; SRH, S-ribosylhomocysteine; LuxS, S-ribosylhomocysteine lyase; AI-2, Autoinducer-2.

In summary, compared to the wild-type and C∆*luxS* strains, the ∆*luxS* strain lacked the ability to produce AI-2 (Figure 3C), leading to reduced abilities in adherence, invasion, autoagglutination, biofilm formation, and stress resistance. Moreover, BA intervention resulted in PCN033 exhibiting the same survival and pathogenicity abilities as those obtained via *luxS* gene deletion. Consequently, BA decreases the pathogenicity of ExPEC by inhibiting the expression level of LuxS, making it more sensitive and easier to eliminate in the host (Figure 9).

## 5. Conclusions

After treatment with BA, the gene expression level of *luxS* in PCN033 was down-regulated, leading to a reduction in the production of AI-2. The decrease in AI-2 production may cause ExPEC to receive inaccurate information, ultimately reducing their ability to resist environmental stress, form a biofilm, perform autoagglutination, and adhere to and invade host cells. The pathogenicity of the ExPEC is diminished by BA, resulting in a higher survival rate of infected mice.

## Figures and Tables

**Table 1:** Bacterial strains and plasmids used in this study.

Strain or Plasmid	Description	Source or Reference
Strain		
PCN033	Wild-type (WT), porcine origin, O11, D Cm^S^	[10]
Δ*luxS*	*luxS* gene mutant strain of PCN033	This study
CΔ*luxS*	The complemented strain of Δ*luxS* of PCN033, Cm^R^	This Study
DH5α	F−, *φ*80dlacZΔM15, Δ(lacZYAargF)U169, deoR, recA1, endA1, hsdR17 (rk−, mk+), phoA, supE44, *λ*−, thi-1, gyrA96, rel1	Takara Bio
*χ*7213	Thi-1 thr-1 leuB6 fhuA21 lacY1 glnV44ΔasdA4 recA1 RP4 2-Tc::Mu[*λ*pir] Km^R^	Dr. Roy Curtiss, USA
plasmid		
pRE112	riT oriV Δasd CmR SacB, suicide vector	Dr. Roy Curtiss, USA
pHSG396	ori lacZ Cm^R^	Takara Bio (Beijing, China)

**Table 2:** List of oligonucleotide primers used in this study.

Primer	Sequence	Remark
*luxS*-u-F	AATTCCCGGGAGAGCTCATACCTTTGAACCGGGTATG(SACI)	Upstream flanking of *luxS*
*luxS*-u-R	AAATTACCGGAGGTGGCTAATCAGTAAACTATCTTCACAATT	
*luxS*-d-F	AATTGTGAAGATAGTTTACTGATTAGCCACCTCCGGTAATTT	Downstream flanking of *luxS*
*luxS*-d-R	TCCCAAGCTTCTTCTAGAGTAAAGATCTGTTCCGCGAT(XBAI)	
Out-Δ*luxS*-F	TATAGTCAACTGGAAGGGCTTG	External source primers for Δ*luxS*
Out-Δ*luxS*-R	GCGCGAAGAGGATTTTGTAG	
In-Δ*luxS*-F	ATGCCGTTGTTAGATAGCTT	Internal source primers forΔ*luxS*
In-Δ*luxS*-R	CTGCAACTTCTCTTTCGGCA	
*luxS*R_F_	CTTCCATTGCCGCTTTCCAG	Primer of *luxS* for q RT-PCR
*luxS*R_R_	TACCCTGGAGCACCTGTTTG	
C*luxS*-F	CGAGGGGTCGACTCTAGAATGCCGTTGTTAGATAGCTTCAC	The sequence of *luxS* with the fragment of plasmid pHSG396
C*luxS*-R	ATTCGAGCTATCGGTACCCTAGATGTGCAGTTCCTGCA	
VF	ATGACCATGATTACGCCAAG	Validation primer for C∆*luxS*
VR	CTACAGCGTGAGCATTGAGAA	
16SRNA_F_	GAATGCCACGGTGAATAC	Primer of 16SRNA for q RT-PCR
16SRNA_R_	GGTTACCTTGTTACGACTTC	

## Data Availability

The data used and/or analyzed during the current study are available from the corresponding author on reasonable request. The data were uploaded to Original data (Yinsheng Qiu Original data [Internet]. Available from: https://doi.org/10.57760/sciencedb.13720, accessed on 27 November 2023).

## References

[1] Smith J.L., Fratamico P.M., Gunther N.W. (2007). Extraintestinal pathogenic *Escherichia coli*. Foodborne Pathog. Dis..

[2] Russo T.A., Johnson J.R. (2000). Proposal for a new inclusive designation for extraintestinal pathogenic isolates of *Escherichia coli*: Expec. J. Infect. Dis..

[3] Troeger H., Richter J.F., Beutin L., Günzel D., Dobrindt U., Epple H.J., Gitter A.H., Zeitz M., Fromm M., Schulzke J.D. (2007). *Escherichia coli* alpha-haemolysin induces focal leaks in colonic epithelium: A novel mechanism of bacterial translocation. Cell. Microbiol..

[4] Totsika M., Kostakioti M., Hannan T.J., Upton M., Beatson S.A., Janetka J.W., Hultgren S.J., Schembri M.A. (2013). A fimh inhibitor prevents acute bladder infection and treats chronic cystitis caused by multidrug-resistant uropathogenic *Escherichia coli* st131. J. Infect. Dis..

[5] Mellata M. (2013). Human and avian extraintestinal pathogenic *Escherichia coli*: Infections, zoonotic risks, and antibiotic resistance trends. Foodborne Pathog. Dis..

[6] Lima-Filho J.V., Martins L.V., Nascimento D.C.d.O., Ventura R.F., Batista J.E.C., Silva A.F.B., Ralph M.T., Vaz R.V., Rabello C.B.V., Da Silva I.d.M.M. (2013). Zoonotic potential of multidrug-resistant extraintestinal pathogenic *Escherichia coli* obtained from healthy poultry carcasses in Salvador, Brazil. Braz. J. Infect. Dis. Off. Publ. Braz. Soc. Infect. Dis..

[7] Ma J., Cheng Z., Bai Q., Zhao K., Pan Z., Yao H. (2021). Screening virulence factors of porcine extraintestinal pathogenic *Escherichia coli* (an emerging pathotype) required for optimal growth in swine blood. Transbound. Emerg. Dis..

[8] Moulin-Schouleur M., Schouler C., Tailliez P., Kao M.R., Brée A., Germon P., Oswald E., Mainil J., Blanco M., Blanco J. (2006). Common virulence factors and genetic relationships between o18:k1:h7 *Escherichia coli* isolates of human and avian origin. J. Clin. Microbiol..

[9] Zhu Y., Dong W., Ma J., Yuan L., Hejair H.M.A., Pan Z., Liu G., Yao H. (2017). Characterization and virulence clustering analysis of extraintestinal pathogenic *Escherichia coli* isolated from swine in china. BMC Vet. Res..

[10] Tan C., Tang X., Zhang X., Ding Y., Zhao Z., Wu B., Cai X., Liu Z., He Q., Chen H. (2012). Serotypes and virulence genes of extraintestinal pathogenic *Escherichia coli* isolates from diseased pigs in China. Vet. J..

[11] Johnson J.R., O’Bryan T.T., Kuskowski M., Maslow J.N. (2001). Ongoing horizontal and vertical transmission of virulence genes and papa alleles among *Escherichia coli* blood isolates from patients with diverse-source bacteremia. Infect. Immun..

[12] Fan Q., Zuo J., Wang H., Grenier D., Yi L., Wang Y. (2022). Contribution of quorum sensing to virulence and antibiotic resistance in zoonotic bacteria. Biotechnol. Adv..

[13] Wu S., Liu J., Liu C., Yang A., Qiao J. (2020). Quorum sensing for population-level control of bacteria and potential therapeutic applications. Cell. Mol. Life Sci. CMLS.

[14] Mayer C., Borges A., Flament-Simon S.C., Simões M. (2023). Quorum sensing architecture network in *Escherichia coli* virulence and pathogenesis. FEMS Microbiol. Rev..

[15] Gopishetty B., Zhu J., Rajan R., Sobczak A.J., Wnuk S.F., Bell C.E., Pei D. (2009). Probing the catalytic mechanism of s-ribosylhomocysteinase (luxs) with catalytic intermediates and substrate analogues. J. Am. Chem. Soc..

[16] Papenfort K., Bassler B.L. (2016). Quorum sensing signal-response systems in gram-negative bacteria. Nat. Rev. Microbiol..

[17] Wang Y., Liu B., Grenier D., Yi L. (2019). Regulatory mechanisms of the luxs/ai-2 system and bacterial resistance. Antimicrob. Agents Chemother..

[18] Li Q., Mao S., Wang H., Ye X. (2022). The molecular architecture of pseudomonas aeruginosa quorum-sensing inhibitors. Mar. Drugs.

[19] Lima E.M.F., Winans S.C., Pinto U.M. (2023). Quorum sensing interference by phenolic compounds—A matter of bacterial misunderstanding. Heliyon.

[20] Huang T., Liu Y., Zhang C. (2019). Pharmacokinetics and bioavailability enhancement of baicalin: A review. Eur. J. Drug Metab. Pharmacokinet..

[21] Tan Y.Q., Lin F., Ding Y.K., Dai S., Liang Y.X., Zhang Y.S., Li J., Chen H.W. (2022). Pharmacological properties of total flavonoids in *Scutellaria baicalensis* for the treatment of cardiovascular diseases. Phytomed. Int. J. Phytother. Phytopharm..

[22] Zhao Q.Y., Yuan F.W., Liang T., Liang X.C., Luo Y.R., Jiang M., Qing S.Z., Zhang W.M. (2018). Baicalin inhibits *Escherichia coli* isolates in bovine mastitic milk and reduces antimicrobial resistance. J. Dairy Sci..

[23] Luo J., Dong B., Wang K., Cai S., Liu T., Cheng X., Lei D., Chen Y., Li Y., Kong J. (2017). Baicalin inhibits biofilm formation, attenuates the quorum sensing controlled virulence and enhances *Pseudomonas aeruginosa* clearance in a mouse peritoneal implant infection model. PLoS ONE.

[24] Peng L.Y., Yuan M., Wu Z.M., Song K., Zhang C.L., An Q., Xia F., Yu J.L., Yi P.F., Fu B.D. (2019). Anti-bacterial activity of baicalin against apec through inhibition of quorum sensing and inflammatory responses. Sci. Rep..

[25] Zong B., Zhang Y., Wang X., Liu M., Zhang T., Zhu Y., Zheng Y., Hu L., Li P., Chen H. (2019). Characterization of multiple type-vi secretion system (t6ss) vgrg proteins in the pathogenicity and antibacterial activity of porcine extra-intestinal pathogenic *Escherichia coli*. Virulence.

[26] Li J., Fan Q., Jin M., Mao C., Zhang H., Zhang X., Sun L., Grenier D., Yi L., Hou X. (2021). Paeoniflorin reduce luxs/ai-2 system-controlled biofilm formation and virulence in *Streptococcus suis*. Virulence.

[27] Zhang B., Ku X., Zhang X., Zhang Y., Chen G., Chen F., Zeng W., Li J., Zhu L., He Q. (2019). The ai-2/luxs quorum sensing system affects the growth characteristics, biofilm formation, and virulence of *Haemophilus parasuis*. Front. Cell. Infect. Microbiol..

[28] Li Z.R., Sun J., Du Y., Pan A., Zeng L., Maboudian R., Burne R.A., Qian P.Y., Zhang W. (2021). Mutanofactin promotes adhesion and biofilm formation of cariogenic *Streptococcus mutans*. Nat. Chem. Biol..

[29] Zhang X., Nakaura Y., Zhu J., Zhang Z., Yamamoto K. (2020). Effect of hyperosmotic salt concentration and temperature on viability of *Escherichia coli* during cold storage. Biocontrol Sci..

[30] Glaubman J., Hofmann J., Bonney M.E., Park S., Thomas J.M., Kokona B., Ramos Falcón L.I., Chung Y.K., Fairman R., Okeke I.N. (2016). Self-association motifs in the enteroaggregative *Escherichia coli* heat-resistant agglutinin 1. Microbiology.

[31] Misawa N., Blaser M.J. (2000). Detection and characterization of autoagglutination activity by *Campylobacter jejuni*. Infect. Immun..

[32] Zong B., Xiao Y., Ren M., Wang P., Fu S., Qiu Y. (2023). Baicalin weakens the porcine expec-induced inflammatory response in 3d4/21 cells by inhibiting the expression of nf-κb/mapk signaling pathways and reducing nlrp3 inflammasome activation. Microorganisms.

[33] Fu S., Yin R., Zuo S., Liu J., Zhang Y., Guo L., Qiu Y., Ye C., Liu Y., Wu Z. (2020). The effects of baicalin on piglets challenged with *Glaesserella parasuis*. Vet. Res..

[34] Ye C., Li R., Xu L., Qiu Y., Fu S., Liu Y., Wu Z., Hou Y., Hu C.A.A. (2019). Effects of baicalin on piglet monocytes involving pkc-mapk signaling pathways induced by *Haemophilus parasuis*. BMC Vet. Res..

[35] Farhadi F., Khameneh B., Iranshahi M., Iranshahy M. (2019). Antibacterial activity of flavonoids and their structure-activity relationship: An update review. Phytother. Res..

[36] Wu S.C., Chu X.L., Su J.Q., Cui Z.Q., Zhang L.Y., Yu Z.J., Wu Z.M., Cai M.L., Li H.X., Zhang Z.J. (2018). Baicalin protects mice against *Salmonella typhimurium* infection via the modulation of both bacterial virulence and host response. Phytomed. Int. J. Phytother. Phytopharm..

[37] Zhang S., Hu B., Xu J., Ren Q., Wang Z., Wang S., Dong Y., Yang G. (2020). Baicalin suppress growth and virulence-related factors of methicillin-resistant *Staphylococcus aureus* in vitro and vivo. Microb. Pathog..

[38] Liu J., Yin F., Liu T., Li S., Tan C., Li L., Zhou R., Huang Q. (2020). The tat system and its dependent cell division proteins are critical for virulence of extra-intestinal pathogenic *Escherichia coli*. Virulence.

[39] Yin F., Hu Y., Bu Z., Liu Y., Zhang H., Hu Y., Xue Y., Li S., Tan C., Chen X. (2023). Genome-wide identification of genes critical for in vivo fitness of multi-drug resistant porcine extraintestinal pathogenic *Escherichia coli* by transposondirected insertion site sequencing using a mouse infection model. Virulence.

[40] Zhu M., Dai X. (2020). Bacterial stress defense: The crucial role of ribosome speed. Cell. Mol. Life Sci. CMLS.

[41] Mihaljevic R.R., Sikic M., Klancnik A., Brumini G., Mozina S.S., Abram M. (2007). Environmental stress factors affecting survival and virulence of *Campylobacter jejuni*. Microb. Pathog..

[42] Thi M.T.T., Wibowo D., Rehm B.H.A. (2020). *Pseudomonas aeruginosa* biofilms. Int. J. Mol. Sci..

[43] Guerra M.E.S., Destro G., Vieira B., Lima A.S., Ferraz L.F.C., Hakansson A.P., Darrieux M., Converso T.R. (2022). *Klebsiella pneumoniae* biofilms and their role in disease pathogenesis. Front. Cell. Infect. Microbiol..

[44] Laird W., Cavanaugh D. (1980). Correlation of autoagglutination and virulence of yersiniae. J. Clin. Microbiol..

[45] Miller M.B., Bassler B.L. (2001). Quorum sensing in bacteria. Annu. Rev. Microbiol..

[46] Yang Q., Wang Y., An Q., Sa R., Zhang D., Xu R. (2021). Research on the role of luxs/ai-2 quorum sensing in biofilm of *Leuconostoc citreum* 37 based on complete genome sequencing. 3 Biotech.

[47] Sztajer H., Lemme A., Vilchez R., Schulz S., Geffers R., Yip C.Y.Y., Levesque C.M., Cvitkovitch D.G., Wagner-Döbler I. (2008). Autoinducer-2-regulated genes in *Streptococcus mutans* ua159 and global metabolic effect of the luxs mutation. J. Bacteriol..

[48] Wang Y., Wang Y., Sun L., Grenier D., Yi L. (2018). The luxs/ai-2 system of *Streptococcus suis*. Appl. Microbiol. Biotechnol..

[49] Ma Y., Hao L., Ke H., Liang Z., Ma J., Liu Z., Li Y. (2017). Luxs/ai-2 in *Streptococcus agalactiae* reveals a key role in acid tolerance and virulence. Res. Vet. Sci..

[50] Stroeher U.H., Paton A.W., Ogunniyi A.D., Paton J.C. (2003). Mutation of luxs of *Streptococcus pneumoniae* affects virulence in a mouse model. Infect. Immun..

[51] Sun Y., Li Y., Luo Q., Huang J., Chen J., Zhang R., Wang X. (2020). Luxs/ai-2 quorum sensing system in *Edwardsiella piscicida* promotes biofilm formation and pathogenicity. Infect. Immun..

